# Quantitative Analysis of Metabolic Abnormality Associated with Brain Developmental Venous Anomalies

**DOI:** 10.7759/cureus.799

**Published:** 2016-09-21

**Authors:** Dmitriy Timerman, Jasmine A Thum, Mykol Larvie

**Affiliations:** 1 Harvard-MIT Division of Health Sciences and Technology, Harvard Medical School; 2 Radiology, Harvard Medical School; 3 Department of Radiology, Massachusetts General Hospital, Boston, MA

**Keywords:** developmental venous anomaly, dva, dva-associated hypometabolism, pet imaging

## Abstract

Background and Purpose: Abnormal hypometabolism is common in the brain parenchyma surrounding developmental venous anomalies (DVAs), although the degree of DVA-associated hypometabolism (DVAAh) has not been quantitatively analyzed. In this study, we demonstrate a simple method for the measurement of DVAAh and test the hypothesis that DVAs are associated with a quantifiable decrement in metabolic activity.

Materials and Methods: Measurements of DVAAh using ratios of standardized uptake values (SUVs) and comparison to a normal database were performed on a cohort of 25 patients (12 male, 13 female), 14 to 76 years old, with a total of 28 DVAs (20 with DVAAh, seven with isometabolic activity, and one with hypermetabolic activity).

Results: Qualitative classification of none, mild, moderate, and severe DVAAh corresponded to quantitative measurements of DVAAh of 1 ± 3%, 12 ± 7%, 18 ± 6%, and 37 ± 6%, respectively. A statistically significant linear correlation between DVAAh and age was observed (P = 0.003), with a 3% reduction in metabolic activity per decade. A statistically significant linear correlation between DVAAh and DVA size was observed (P = 0.01), with a 4% reduction in metabolic activity per each 1 cm in the longest dimension. The SUV_DVA_-based measures of DVAAh correlated (P = 0.001) with measures derived from comparison with a standardized database.

Conclusion: We present a simple method for the quantitative measurement of DVAAh using ratios of SUVs, and find that this quantitative analysis is consistent with a qualitative classification. We find that 54% (15 of 28) of DVAs are associated with a greater than 10% decrease in metabolic activity.

## Introduction

Developmental venous anomalies (DVAs) are the most common vascular malformation identified on brain imaging [1]. Traditionally, DVAs have been considered benign in that they are not commonly associated with symptoms, and evidence suggested a low frequency of adverse effects [2]. However, several studies report an association between MRI signal abnormalities within brain parenchyma in the drainage territory of 12.5% [3] to 28.3% [4] of DVAs in adults. These studies show an increased prevalence of MR signal abnormalities in older patients, which raises the possibility of increasing brain parenchymal abnormality with age that may then become symptomatic. Possible mechanisms of DVA pathophysiology include venous congestion [5], hypoperfusion [6], and venous hypertension leading to ischemia and microhemorrhage [7-8]. Multiple imaging examples of parenchymal and perfusion abnormalities associated with DVAs have been documented [9]. We previously showed the presence of a mild, moderate, or severe degree of DVA-associated hypometabolism (DVAAh) in 72% (18 of 25) of the DVAs in patients who had a known DVA and FDG-PET imaging [10]. A quantitative analysis of DVAAh, however, has not been reported. In this study, we present a method for the measurement of DVAAh and test the hypothesis that DVAs are associated with a quantifiable decrement in metabolic activity that is associated with DVA size and patient age.

## Materials and methods

### Patient population

A total of 25 patients with 28 distinct DVAs were included for analysis in this study. A summary of the DVA characteristics and associated findings is presented in Table 1. In a prior study [10], we reported on 22 of these patients with a total of 25 DVAs, which are now included in the current study as well as three additional cases. The prior study was a qualitative analysis based on visual inspection. This current study employs a quantitative analysis using methods that are easily employed in clinical practice. This study was performed at a tertiary care academic medical center for adult and pediatric patients and was approved by the Partners Healthcare Institutional Review Board (approval #2013P001673). Informed patient consent was obtained at the time of treatment. Criteria for inclusion in the study were the following: 1) patients with at least one cerebral or cerebellar DVA identified by MRI and reconfirmed by a neuroradiologist; 2) positive confirmation of the DVA, as defined by the presence of radially converging dilated medullary veins (caput medusa) converging to a large transparenchymal vein that drains into a deep or superficial vein; and 3) availability of an FDG-PET scan performed within one year of the MRI examination demonstrating the DVA. Cases were excluded if any of the following criteria were present: 1) PET or MRI findings suggestive of a neurodegenerative disease, or 2) a confounding lesion, such as a tumor, in the region of the DVA or the anatomically equivalent contralateral cortex.

**Table 1:** Summary of Characteristics of All Patients and DVAs* *Each DVA was counted separately in the three patients with multiple DVAs. Twenty-two of these 25 patients were qualitatively presented in a prior study [10].

Summary of Characteristics of All Patients and DVAs*​	
Characteristic	
Total No. of patients	25
No. of DVAs per patient	
1	22 (88%)
2	3 (12%)
Age (years)	
Mean ± SD	44.3 ± 19.4
Range	14 - 76
Male, female sex	12 (48%), 13 (52%)
Degree of DVA-associated metabolic activity	
Isometabolic	7 (25%)
Hypometabolic	20 (71%)
Mild	12 (43%)
Moderate	5 (18%)
Severe	3 (11%)
Hypermetabolic	1 (4%)
Mild	0 (0%)
Moderate	0 (0%)
Severe	1 (4%)

### MR imaging

The included MR examinations were performed using a variety of imaging protocols that were prescribed according to the clinical indication for the scan and have been described previously [10]. Briefly, all MR imaging was performed at our institution using 1.5T (GE and Siemens), 3.0T (GE and Siemens), and 7T (Siemens) scanners (General Electric Co., Boston, MA ) (Siemens Healthcare, Malvern, PA). Post-contrast T1-weighted images, T2 images, and susceptibility-weighted imaging (SWI) images were preferentially used for evaluation and confirmation of the DVA.

### 18F-fluorodeoxyglucose positron emission tomography (18F-FDG-PET) imaging

All PET imaging was performed at our institution using a standard protocol as described previously [10]. Briefly, imaging was obtained approximately 45 minutes following intravenous injection of 5.0 mCi of 18F-fluorodeoxyglucose (FDG) using an ECAT HR+ scanner (Siemens/CTI, Knoxville, TN) and 63 planes were acquired simultaneously over a 15.5 cm field of view in 3D mode. Attenuation correction was performed using a transmission scan obtained with a 68Ge source, and a maximum likelihood reconstruction method was used, yielding images with an in-plane resolution of approximately 4.6 mm. All patients were screened with fingerstick blood glucose measurement; PET imaging was not performed in those patients with blood glucose greater than 150 mg/dl.

### Image analysis

The MR and PET images were co-registered using Syngo.via and Syngo TrueD systems (Siemens Healthcare, Malvern, PA) software, which was then used for the measurement of the average standardized uptake value (SUV) in a 1 cm^3^ spherical region of interest (ROI) around the visually determined lesions and in the anatomically equivalent contralateral brain region. The SUV represents the radioactivity concentration observed in an ROI relative to the hypothetical case of homogeneous distribution of the injected radioactivity across the entire body of the patient. In this study, SUV_DVA_ is defined as the mean SUV from the region of the DVA divided by the mean SUV in the anatomically equivalent contralateral region. The size of each DVA was measured as the single longest dimension of the aberrant vasculature. The qualitative characterization of the degree of metabolic abnormality (mild, moderate, or severe hypometabolism; isometabolism; or mild, moderate, or severe hypermetabolism) was determined by a consensus of the authors.

### Database comparison

**Figure 1:**
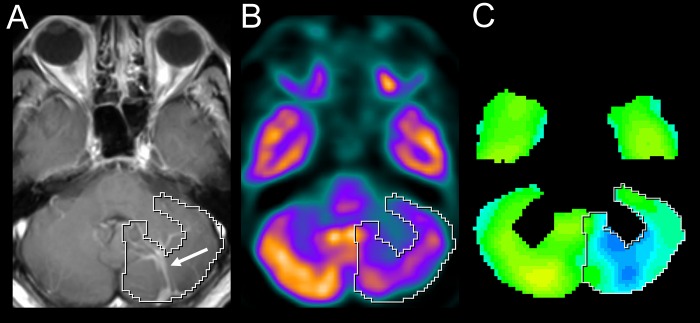
Representative axial MR (A), FDG-PET (B), and statistical map (C) of DVA-affected brain. A) Post-contrast T1 image shows a DVA (white arrow) within the left cerebellar hemisphere. B) FDG-PET demonstrates the DVAAh, qualitatively classified as severe, that corresponds to an SUVR of 0.65. C) The statistical map graphically depicts the standard deviations of the metabolic activity relative to a normal subject database. In this case, the auto-segmented region (white outline) of the left cerebellum corresponds to -3.6 standard deviations from the mean SUV in this region for normal controls.

All PET studies were compared to a normal control patient database with Syngo.via software using the Molecular Imaging Neurology workflow (Figure 1). The PET images were aligned with an ensemble-average image derived from the normal database and automatically segmented. A region corresponding to the DVA was manually selected in each case and the difference in standard deviations compared to a normal database was recorded. Separate comparison databases were used for younger subjects (age: 19-44) and older subjects (age: 46-79).

### Statistical analysis

Statistical analyses were performed using MATLAB (The MathWorks, Inc., Natick, MA, USA). Differences less than the threshold of α = 0.05 were considered statistically significant. Analysis of variance was used to identify statistically significant differences in SUV_DVA_ among the previously qualitatively-labeled groups. A linear regression model was used to determine if there was a statistically significant correlation between SUV_DVA_ and patient age, SUV_DVA_ and DVA size, and SUV_DVA_ and the standard deviation from the mean SUV derived from a database of normal subjects.

## Results

**Figure 2:**
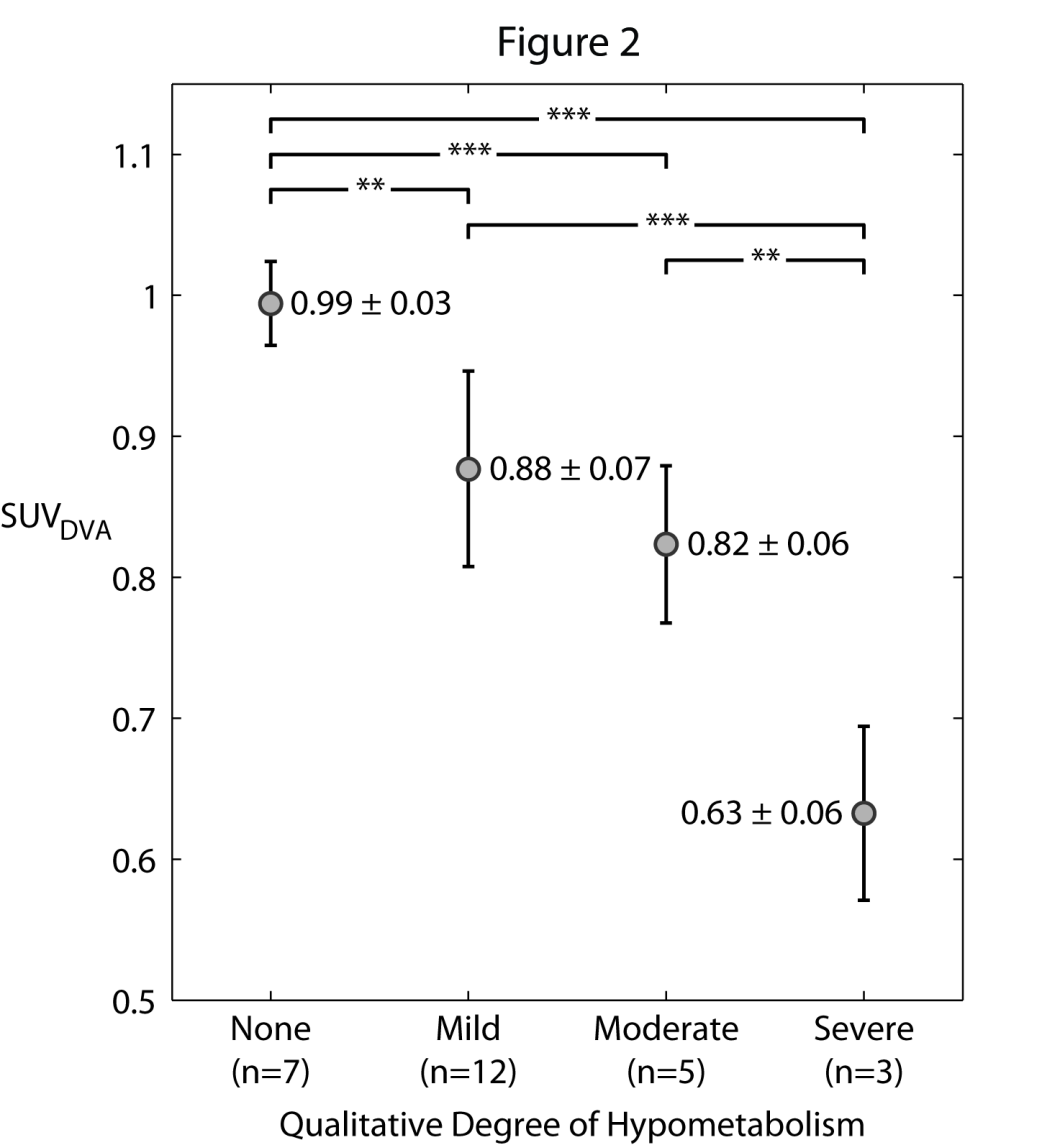
SUV of DVA measurements for qualitative DVAAh classification. The SUV_DVA_ was determined based on the mean SUV from the cortex drained by the DVA divided by the mean SUV from the approximated anatomically equivalent region in the contralateral brain. The previously defined “none”, “mild”, “moderate”, and “severe” qualitative degree of DVAAh correspond to a 1 ± 3%, 12 ± 7%, 18 ± 6%, and 37 ± 6% decrease, respectively, in the SUV measured from the region of DVAAh compared to the contralateral region. Asterisks indicate statistical significance: *P < 0.01, **P < 0.001, ***P < 0.0001.

The qualitative classification of none, mild, moderate, and severe DVAAh corresponded to a mean ± standard deviation of 1 ± 3%, 12 ± 7%, 18 ± 6%, and 37 ± 6% decrease, respectively, in the SUV measured on the side of the DVA compared to the anatomically equivalent contralateral region. As shown in Figure 2, the mean SUV_DVA_ for the severe DVAAh category was found to be significantly different from the mean SUV_DVA_ of all other qualitative DVAAh designations (none and mild: P < 0.0001; moderate: P < 0.01). The mean SUV_DVA_ for no DVAAh was also found to be significantly different from the mean SUV_DVA_ of all other qualitative DVAAh designations (mild: P < 0.001; moderate and severe: P < 0.0001). There was no statistical difference in the quantitative measures associated with moderate and mild DVAAh (P = 0.1).

**Figure 3:**
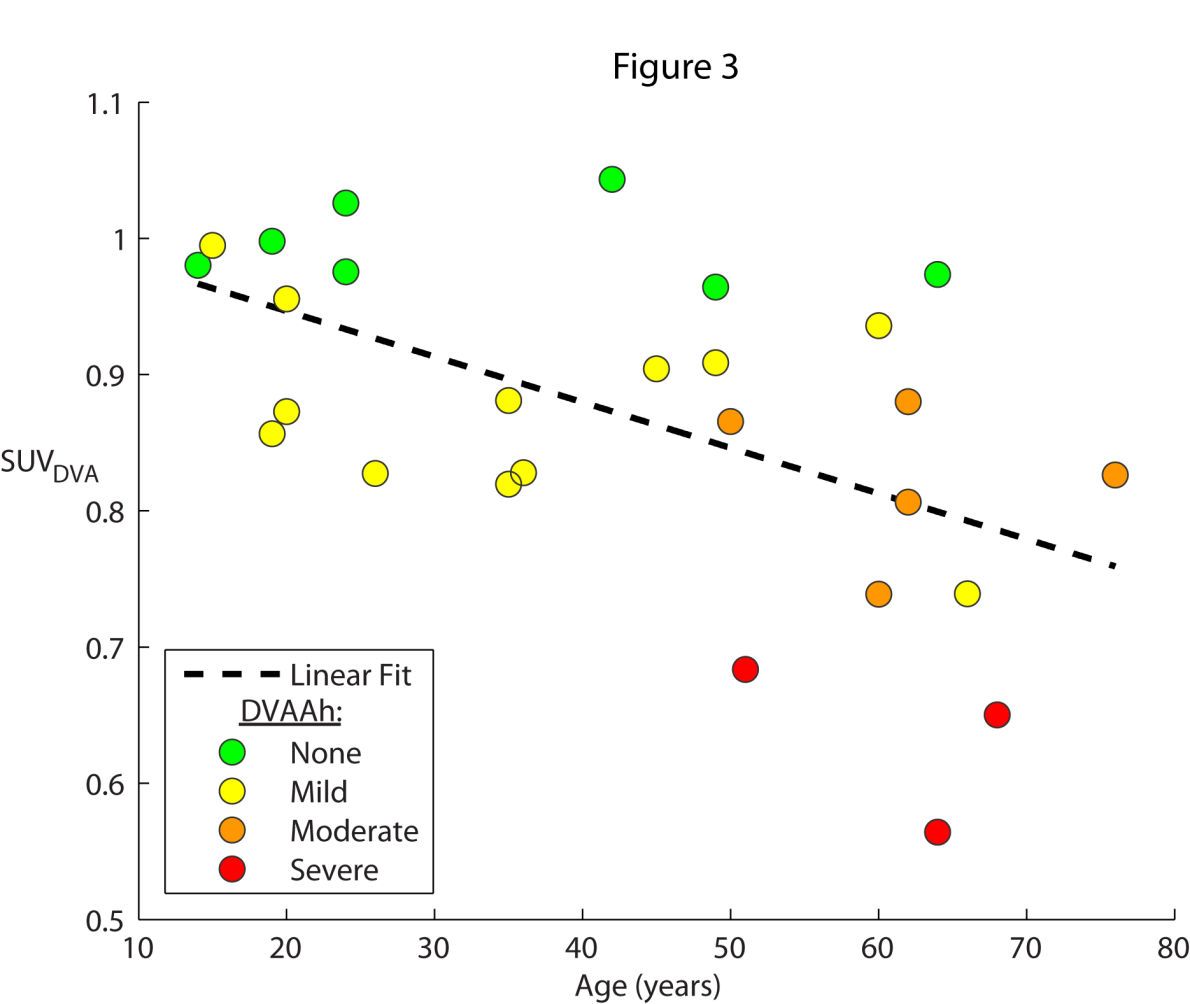
SUV of DVA compared to patient age The degree of DVAAh compared to the contralateral anatomic region (SUV_DVA_) were plotted as a function of age (in years) for each DVA in the series. A linear regression (SUV_DVA_ = -0.003 * Age (years) + 1.0) was fit to the data, with R^2^ = 0.29; P = 0.003. No moderate (orange) or severe (red) cases were seen in patients less than 50 years of age. The color of each point indicates the qualitative degree of DVAAh compared to the contralateral anatomic region (none: green; mild: yellow; moderate: orange; severe: red).

**Figure 4:**
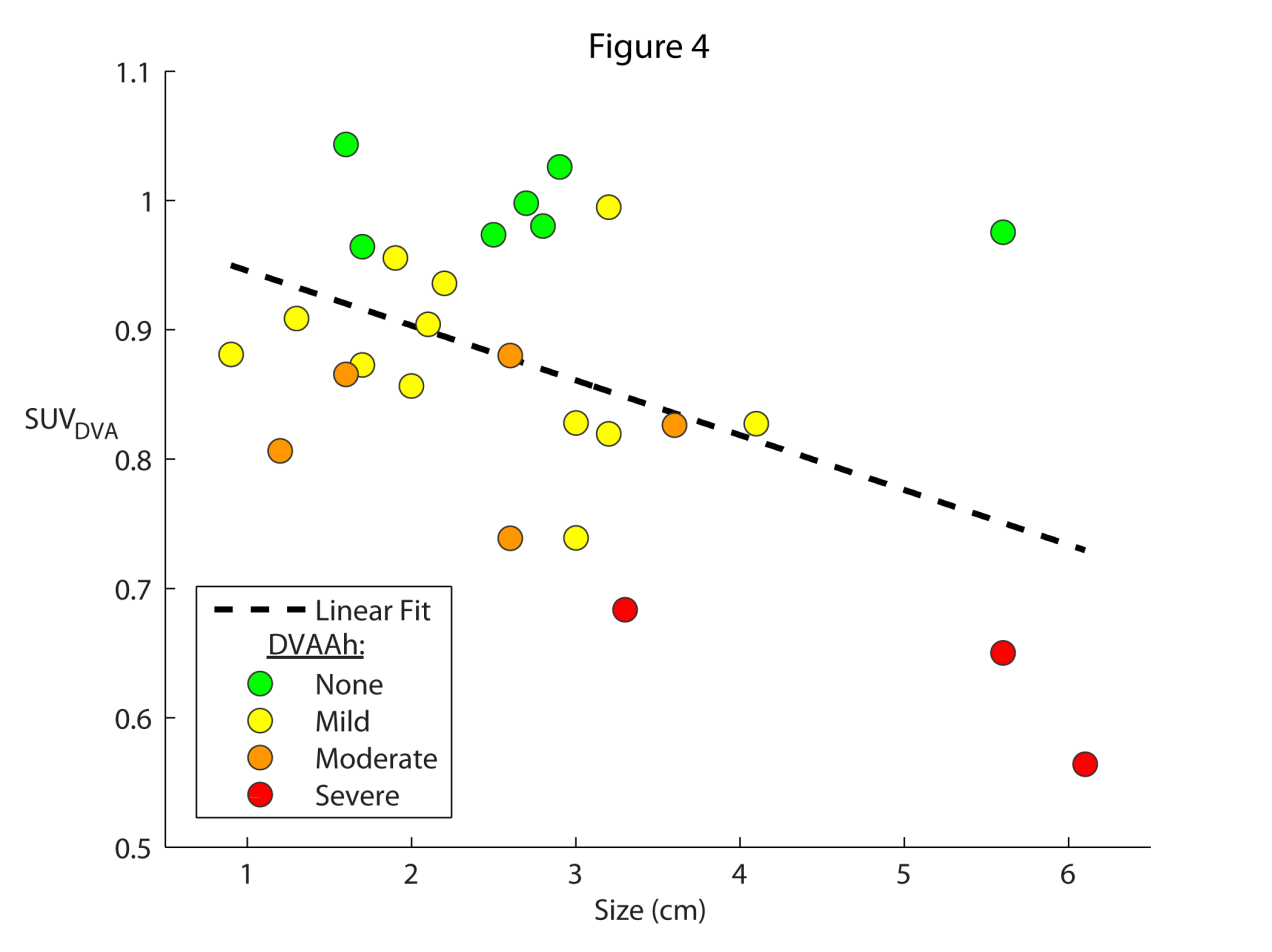
SUV of DVA compared to DVA size. The degree of DVAAh compared to the contralateral anatomic region (SUV_DVA_) were plotted as a function of size (in cm) for each DVA. A linear regression (SUV_DVA_ = -0.04 * Size (in cm) + 0.99) was fit to the data, with R^2^ = 0.22; P = 0.01. The color of the plot point indicates the qualitative degree of hypometabolism of the DVA compared to the contralateral brain region (none: green; mild: yellow; moderate: orange; severe: red). The outlier measuring 5.6 cm in the “none” group was not removed from the regression analysis and corresponds to a 24-year-old patient.

**Figure 5:**
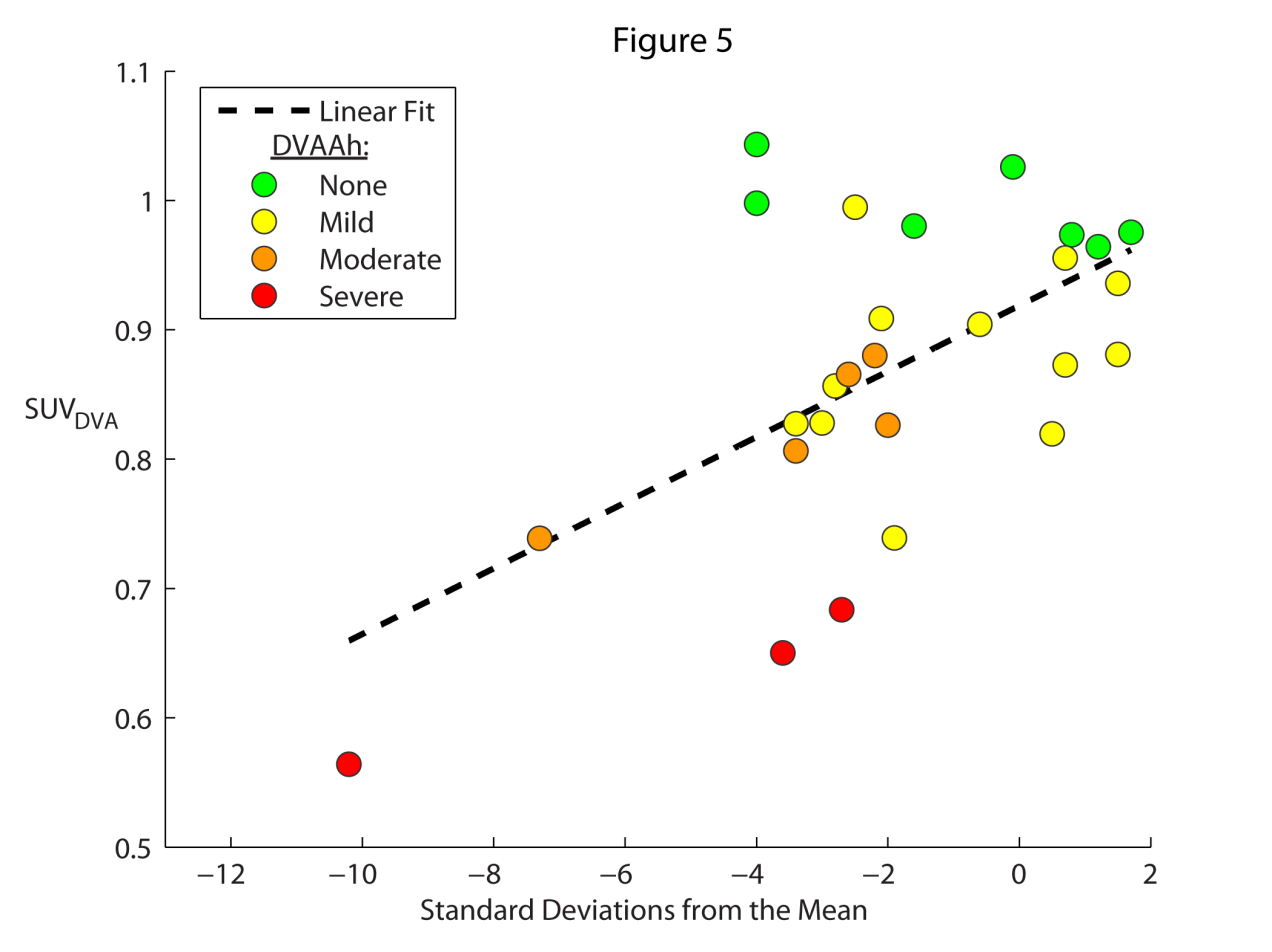
SUV of DVA compared to standard deviations from the mean. The degree of DVAAh compared to the contralateral brain region (SUV_DVA_) was plotted as a function of the standard deviations from the normal mean (SD) for each DVA. A linear regression (SUV_DVA_ = 0.02 * SD + 0.92) was fit to the data, with R^2^ = 0.34; P = 0.001. The color of the plot point indicates the qualitative degree of hypometabolism of the DVA compared to the contralateral anatomic region (none: green; mild: yellow; moderate: orange; severe: red).

Figures 3-5 depict all of the cases color-coded based on the qualitative DVAAh designations: green for none, yellow for mild, orange for moderate, and red for severe. The cases of no (green) DVAAh are centered on an SUV_DVA_ of approximately one while the severe (red) DVAAh cases are found only at an SUV_DVA_ level of less than 0.7. In the one previously reported patient who demonstrated severe DVA-associated hypermetabolism [10], the SUV_DVA_ in the hypermetabolic ROI was 3.6 (this anomalous case was not included in Figures 2-5 or any correlation analysis in this study).

A statistically significant (P = 0.003, R^2 ^= 0.29) linear correlation between SUV_DVA_-based DVAAh and age was observed, with a 3% rate of decline in relative metabolic activity per decade (Figure 3). Moderate (orange) or severe (red) cases were only seen in patients older than 50 years.

As shown in Figure 4, a statistically significant (P = 0.01, R^2 ^= 0.22) linear correlation between SUV_DVA^-^_based DVAAh and DVA size was observed, with a 4% rate of decline in metabolic activity for each 1 cm in the longest dimension (lesions ranged between 0.9 and 6.1 cm long). Within the no DVAAh (green) subgroup, six of seven DVAs were less than 3 cm in greatest dimension, with a notable outlier measuring 5.6 cm. This patient with a 5.6 cm DVA was 24 years old. The mild (yellow) and moderate (orange) DVAAh cases ranged in size from < 1 cm to 4 cm, and all severe (red) DVAAh cases were > 3 cm (Figure 4). There was no statistically significant association between DVA size and patient age (P = 0.52, data not shown).

There is a statistically significant (P = 0.001, R^2 ^= 0.34) linear correlation between SUV_DVA_ and the standard deviation from the mean SUV derived from a database of normal subjects (Figure 5).

## Discussion

In this study, we describe a quantitative assessment of the degree of DVAAh, performed using a method that may be employed in clinical practice. We used an SUV_DVA_ analysis to compare DVAAh between patients under the assumption that the injected radioactivity and body weight components of the SUV formula are canceled out [11-13]. This method allowed us to quantify the degree of DVAAh and quantitatively assess the visual classifications of mild, moderate, and severe from our prior study. An advantage of using the SUV_DVA_ as an objective measure of altered metabolic activity in the drainage region of a DVA is the relative simplicity of this analysis and consequent potential for clinical application of this method. Measurement of the SUV_DVA_ can be used to confirm a qualitative assessment of the degree of change in metabolic activity in the region of a DVA. This quantitative measurement of DVAAh can be combined with the patient’s clinical history to aid in the determination of how a DVA should be managed.

The worsening of DVAAh with increasing age could imply that the natural history of DVAs may result in either increasing damage to adjacent brain parenchyma or possibly remodeling of parenchyma away from the region with altered venous drainage. We find that no patients under the age of 50 years old had more than a 20% reduction in SUV_DVA_, which complements findings of increased MR abnormalities associated with DVAs in patients of older age in prior studies [3-4]. However, the SUV_DVA_ measurement of DVAAh in patients over 50 years old ranged from severely decreased to nearly normal (0.56 to 0.97), suggesting that some DVAs tend to have an increased degree of DVAAh than others. A prospective, longitudinal study would be ideal to determine if progression of DVAAh exists on an individual patient level. Such an analysis would require performing repeat FDG-PET imaging to measure the relatively small rate of change in DVAAh, which, according to our data, is on the order of 3% per decade. Further research is also necessary to determine if the suggested 50-year-old age threshold contributes to the identification of patients in whom there is clinical concern that a DVA may be symptomatic.

Additional studies could also clarify if particular DVA morphology may predict the degree and progression of DVAAh independent of patient age. One study of pediatric patients suggested that the presence of deep venous drainage in DVAs may play an independent role in the prediction of associated brain parenchymal signal abnormality [14].

Although there is an inverse relationship between DVAAh and DVA size in our data, there were no clear thresholds between any of the qualitative groups based on size. This may indicate that the measurement of the longest DVA dimension is not an optimal index of true DVA size, the parameters for SUV_DVA_ measurement could be redefined to more accurately represent DVAAh, and/or that there are additional features and physiologic mechanisms that can contribute to better prediction of which DVAs are associated with greater DVAAh than others.

Our results did not show a significant correlation between DVA size and patient age. However, our analysis was limited to a retrospectively defined cohort of patients with PET imaging, as this study was not designed to analyze for DVA growth. Consequently, the question of whether or not DVAs enlarge with age remains to be answered. A comparison of DVA size to patient age does not require the presence of PET imaging. A study testing such an association would likely identify some patients with multiple longitudinal MR scans, enabling individualized size comparisons over time. Of note, one 24-year-old patient with a DVA of 5.6 cm demonstrated no DVAAh, which may be attributable to the relatively young age.

We also performed statistical parametric mapping in order to compare the difference in metabolic activity in our cohort of patients compared to a database. This was done using software that auto-aligned and auto-segmented the PET data for each patient, and subsequently, the region encompassing the DVA was manually selected. Although the auto-alignment and auto-segmentation of brain regions were objective, the manual selection of the region corresponding to the DVA was occasionally challenging given that some DVAs did not respect standard anatomical divisions and DVAAh was, in some cases, represented in two or more neighboring segments. Furthermore, for small DVAs, the segmented regions were generally larger than the region affected with DVAAh; consequently, the measured number of standard deviations from the baseline was underestimated by the inclusion of unaffected brain parenchyma. However, despite these limitations, the correlation between this automated measure of DVAAh and the SUV_DVA_-based measure of DVAAh was statistically significant.

There are several limitations of this study. The number of cases analyzed was limited by the scarcity of patients with DVAs who also had FDG-PET imaging. To develop a robust measure of the incidence of DVAAh, it will be necessary to reproduce these results in other patient cohorts. The greater incidence of metabolic derangement and co-morbidities, particularly in older patients, is a potential confounder that could contribute to the observed increase in DVAAh severity. There are also limitations to the SUV_DVA_ method for quantifying DVAAh [13]. Manual selection of ROIs results in individually-biased results that may have suboptimal reproducibility. To partially avoid user-bias, neuroradiologists can select ROIs on an MR scan of the patient prior to registration with the PET study. Robust inter-user reproducibility has been shown in other studies that specifically compared manual and automated selection of SUV ROIs [15]. Image noise and limited resolution also affect the SUV measurement. Another limitation of this study is the technique used to determine DVA size. The measurement for DVA size was an estimate based on the largest dimension of the DVA in any plane. This approach to characterizing DVA size is of limited accuracy and is not a robust assessment of the diversity of DVA morphology. However, in the clinical setting, it is a practical method for estimating DVA size. Overall, this quantitative approach could be a clinically useful supplement to the visually-determined qualitative assessment of DVAAh. 

## Conclusions

We find that quantitative analysis of DVAAh using SUV_DVA_ is consistent with a visual, qualitative assessment of DVAAh. We find that 54% (15 of 28) of DVAs are associated with a greater than 10% decrease in metabolic activity as measured by SUV_DVA_ measured from FDG-PET. These results show that simple, quantitative measurements may be reliably used for evaluation of metabolic abnormality associated with DVAs and may be helpful in classifying patients that may warrant further examination or intervention. While we have demonstrated that DVAs are associated with measurable hypometabolic activity, the short and long-term clinical implications of this finding are not well understood. Further investigation into the natural history of DVAs, including the evolution of DVAAh, is necessary to determine the appropriate management of patients with DVAs.
